# A Comprehensive Case Report Emphasizing the Role of Caesarean Section, Antibiotic Prophylaxis, and Post-operative Care in Meconium-Stained Fetal Distress Syndrome

**DOI:** 10.7759/cureus.66123

**Published:** 2024-08-04

**Authors:** Arun E., Nirenjen S., Arunkumar Subramanian, Tamilanban T., Chitra Vellapandian, Narayanan J.

**Affiliations:** 1 Pharmacology and Therapeutics, SRM College of Pharmacy, SRM Institute of Science and Technology, Chennai, IND; 2 Pharmacology, SRM College of Pharmacy, SRM Institute of Science and Technology, Chennai, IND; 3 Pharmacy/Pharmacology, SRM College of Pharmacy, SRM Institute of Science and Technology, Chennai, IND

**Keywords:** amniotic fluid, fetal distress, lower segment caesarean section (lscs), antibiotic prophylaxis, meconium aspiration syndrome (mas)

## Abstract

Meconium-stained amniotic fluid (MSAF) presents a complex medical scenario with significant implications for maternal and neonatal health. This case report explores the intricacies surrounding MSAF, focusing on its diagnosis, treatment, and the associated meconium aspiration syndrome (MAS). The report emphasizes the critical role of antibiotic prophylaxis in lower segment cesarean sections (LSCS) in balancing infection prevention in the mother with neonatal considerations. Additionally, it highlights personalized pain management and post-operative care regimens, contributing to a comprehensive strategy for maternal and neonatal well-being.

A 27-year-old primigravida (primi) underwent a cesarean section due to the presence of meconium in the amniotic fluid, indicating fetal distress. The report meticulously documents vital signs, laboratory findings, and the timeline of events. The case report underscores the importance of diagnosing and treating MAS, offering valuable insights into management strategies and their impact on maternal and neonatal health. This case report emphasizes the critical role of antibiotic prophylaxis in LSCS to prevent maternal infection while considering neonatal well-being. The personalized pain management approach and post-operative care regimens contribute significantly to a comprehensive strategy for maternal and neonatal well-being. The findings provide valuable insights into diagnosing and treating MAS, highlighting the importance of timely intervention in similar clinical scenarios.

## Introduction

The presence of a green hue in the amniotic fluid, caused by the presence of meconium, is a common concern during pregnancy. Meconium is a dense, green-black substance originating from the fetal colon [1,2]. It is composed of water and is a mixture of elements, including skin cells, intestinal and urinary tract cells, lanugo hair, fatty material known as vernix caseosa, and digestive secretions, particularly bile, which impart the distinctive green color. The presence of meconium in the amniotic fluid serves as a signal of fetal distress and is associated with an elevated risk of perinatal health complications. Meconium-stained amniotic fluid (MSAF) often leads to more intricate deliveries, with higher occurrences of interventions such as cesarean sections, labor induction, neonatal intensive care unit (NICU) stays, and neonatal mortality [3]. Meconium aspiration syndrome (MAS) occurs in approximately 2% to 10% of MSAF cases, and it arises when the baby inhales meconium [4,5]. The introduction of fetal meconium into the amniotic fluid typically happens when the fetus experiences oxygen deprivation, either acutely or chronically [6].

Several factors can contribute to the occurrence of MSAF, including maternal drug use or abuse (such as cocaine or tobacco), pre-eclampsia, placental insufficiency, oligohydramnios, and post-term pregnancies [7,8]. Meconium passage is infrequent before 34 weeks of pregnancy but becomes more common after 37 weeks. The presence of MSAF during pregnancy significantly elevates the risk of respiratory distress in infants, approximately 100 times higher than in cases with clear amniotic fluid. Meconium aspiration, which is observed in about 20-30% of newborns with MSAF, takes place when meconium is found below the vocal cords. This can occur when the fetus gasps in utero or during a baby's initial breaths after birth [9,10]. MAS is marked by respiratory distress soon after birth, the presence of MSAF, and indications of aspiration pneumonitis on radiographic examinations. Fortunately, stillbirth rates have recently decreased due to advances in prenatal and postpartum care, resulting in improved newborn outcomes [11,12]. In comparison to vaginal delivery, a caesarean delivery is associated with a fivefold increase in the risk of maternal infectious complications. However, the utilization of prophylactic antibiotics can significantly enhance the outcomes of caesarean deliveries, mitigating the risk of such complications [13,14].

This case report aims to investigate the impact of MSAF on perinatal health, exploring associated risks, interventions, and outcomes. It also seeks to identify maternal and fetal factors contributing to MSAF, offering insights for prevention and management, including considerations for delivery methods and addressing maternal infectious risks.

## Case presentation

A 27-year-old female patient was admitted to the obstetrics and gynecology department in a tertiary care teaching hospital (SRM Medical College Hospital and Research Institute) with chief complaints of primigravida (primi)/term pain since morning. Her medical history revealed that she was in her third trimester of pregnancy and is currently able to perceive fetal movements. She had no known complaints of diabetes mellitus, hypertension, bronchial asthma, coronary artery disease, or hypothyroidism. There was no history of past medication use, and she had not undergone any previous surgeries. Her last menstrual period (LMP) was on April 28, 2019. In terms of obstetrical history, she was a primi. The patient maintains a mixed food diet pattern with normal appetite, sleep patterns, and bowel and bladder habits.

In the course of the patient's hospitalization, i.e., eight days (January 21, 2020-January 28, 2020), a series of vital signs were diligently recorded. On the initial date of January 21, the patient was found to be afebrile with a pulse rate (PR) of 78 bpm and a respiratory rate (RR) of 20 bpm. Notably, her blood pressure (BP) was measured at 120/180 mmHg. Over the subsequent days, from January 22 to January 26, her temperature remained within the normal range, being consistently afebrile. Her PR remained steady at 78 bpm, and her RR ranged from 20 to 22 bpm. There was some variation in her BP, with readings fluctuating between 110/70 and 120/180 mmHg. These recorded vital signs serve as essential data points for the ongoing assessment and care of the patient and are discussed in Table 1.

**Table 1:** Patient Vitals Afebrile temperature indicates absence of fever; PR (bpm): Pulse rate in beats per minute; RR (bpm): Respiratory rate in breaths per minute; BP (mmHg): Blood pressure in millimeters of mercury, with the first value representing systolic pressure and the second diastolic pressure.

Vitals	Dates					
	21/01	22/01	23/01	24/01	25/01	26/01
Temperature	Afebrile	Afebrile	Afebrile	Afebrile	Afebrile	Afebrile
PR (bpm)	78	78	70	70	70	78
RR (bpm)	20	22	20	22	20	20
BP (mmHg)	120/180	110/80	110/90	120/80	120/80	110/70

The cardiovascular system exhibited normal heart sounds with no detected murmurs upon system examination. The respiratory system showed no unusual findings. The abdomen displayed soft tissue consistency with no presence of masses or tenderness. In the central nervous system, there were no discernible focal neurological deficits.

Laboratory findings

The laboratory investigation results revealed several critical parameters for the patient. The hemoglobin (Hb) level was measured at 12.0 g/dL, falling within the normal range of 13-18%. The packed cell volume (PCV) was slightly lower at 29%, below the expected range of 35-49%. The blood urea level was 22 mg/dL, marginally elevated compared to the normal range of 7-20 mg/dL, indicating potential kidney function concerns. Serum creatinine (Sr.Cr) was measured at 0.5 mg/dL, which was below the upper limit of the standard range (0.7-1.2 mg/dL), suggesting good renal function. Glucose levels were assessed with random blood sugar (RBS) at 94 mg/dL, within the expected range of 80-160 mg/dL. The fasting blood sugar (FBS) was notably low at 60 mg/dL, well below the recommended threshold of 100 mg/dL, possibly indicating hypoglycemia. Postprandial blood sugar (PPBS) was 116 mg/dL, still comfortably below the threshold of 140 mg/dL. Thyroid function markers show free thyroxine (FT4) at 1.34 mg/dL, indicating an average thyroid hormone level within the range of 0.7-2.0 mg/dL. Free triiodothyronine (FT3) was 3.19 pg/mL, within the normal range of 2.3-4.3 pg/mL. Thyroid stimulating hormone (TSH) was measured at 1.51 µIU/mL, suggesting the thyroid functions generally within the range of 0.3-5.0 µIU/mL. In summary, the laboratory investigation suggested that the patient had a slightly lower PCV and elevated blood urea, which required further evaluation. However, other parameters like glucose and thyroid function appeared to be within the normal range, as discussed in Table 2.

**Table 2:** Laboratory Investigations Hb: Hemoglobin; PCV: Packed cell volume; Sr.Cr: Serum creatinine; RBS: Random blood sugar; FBS: Fasting blood sugar; PPBS: Postprandial blood sugar; FT4: Free thyroxine; FT3: Free triiodothyronine; TSH: Thyroid stimulating hormone

Parameter	Diagnosed Value	Normal Range
Hb	12	13-18%
PCV	29	35-49%
Blood urea	22	7-20
Sr.Cr	0.5	0.7-1.2
RBS	94	80-160mg/dl
FBS	60	<100mg/dL
PPBS	116	<140mg/dL
FT4	1.34	0.7-2.0mg/dL
FT3	3.19	2.3-4.3pg/mL
TSH	1.51	0.3-5.0 µIU/mL

Overview of prenatal care and monitoring

The particular investigation findings include information related to pregnancy, with specific details and impressions provided.

*Early Scan* 

The early scan results indicate that the pregnancy was at seven weeks and six days of gestation. This was an essential milestone in monitoring the development of the fetus during the first trimester and was found to be normal.

*Nuchal Translucency (NT) Scan* 

The NT scan is typically performed around 12 to 14 weeks of gestation. This scan helps assess the risk of specific chromosomal abnormalities in the developing fetus, and No abnormality was detected in this case.

Scan Estimated Due Date (EDD)

The EDD calculated from the scan results was January 21, 2020, which was slightly earlier than the EDD.

Date Reference

On January 7, 2020, the pregnancy was at 37 weeks and 3 days. This date reference may be essential for tracking the progression of the pregnancy.

*EDD* 

The EDD calculated to be January 25, 2020. This was the expected date when the baby likely to be born. In this case, patient experienced labor on January 21, 2020.

From anticipation to adaptation: Caesarean section (lower segment cesarean section (LSCS))

Monitoring labor and making decisions regarding the necessity of interventions during the patient's admission to the obstetrics and gynecology department for childbirth on January 21, 2020, relied on crucial factors such as the cervix's dilation and effacement, the baby's positioning, and the condition of the amniotic fluid. Pertinent information was gathered through vaginal examinations at various time points.

12:30 pm

The cervix was 25% effaced, meaning it has started to thin out, and The OS (cervical opening) admits the tips of a finger, indicating some initial dilation. The baby's head was above the brim of the pelvis.

12:45 pm

The cervix was now 50% effaced, indicating further thinning. The cervical OS was 2 cm dilated, meaning the cervix had opened to 2 cm. Membranes were ruptured (membrane+), which means the amniotic sac had broken. The baby's presentation (Vx) was at -3, which indicated the baby's position in the pelvis. The pelvis was described as adequate, suggesting it was suitable for labor progress.

1:15 pm

Membranes were reported to have “thick meconium-stained liquor." This suggests that the amniotic fluid was discolored and contains meconium, a sign of fetal distress.

MAS

Meconium aspiration observed served as a sign of fetal distress, potentially posing harm to the fetus. The healthcare team promptly opted for a caesarean section (LSCS) as a safer method of delivering the baby. The primary aim of this surgical procedure was to prevent the newborn from potentially inhaling meconium, which, if inhaled, could result in respiratory complications and other health issues. The healthcare professionals carefully assessed the circumstances and made a well-informed decision regarding the most appropriate course of action for ensuring the well-being of both the mother and the baby when confronted with MAS. The specific choice of intervention was determined by various factors, including the severity of the situation and the overall health status of both the mother and the baby.

Treatment

Pre-operative Notes

This section includes ensuring the patient's NPO (nothing by mouth) status and obtaining written informed consent.

Intravenous medications were administered as follows: Inj. ranitidine (2 ml) and Inj. ondansetron (2 ml) were provided on a STAT (one-time immediate dose) basis, ensuring that the latter was administered half an hour prior to the surgical intervention. Additionally, a STAT order of IV fluid Ringer's lactate (1 pint) was administered to maintain the patient's hydration status. To assess the patient's tolerance to local anesthesia, a test dose of Inj. xylocaine (0.5 ml) was administered, and, as a prophylactic measure against infection, Inj. cefotaxime (1 g) was administered on a STAT basis one hour before surgery.

Post-operative Notes

In the post-operative care for patients, the following medication regimen was diligently administered. For essential nutritional support and overall well-being, the patient received a BCT tablet (B-complex), FST tablet (ferrous sulphate), and calcium tablet, all taken orally once daily. IV hydration was provided with one pint of IV fluid Ringer's lactate daily, with a brief interruption on the 28th day. In comparison, two pints of IV fluid normal saline (NS) and one pint of IV fluid dextrose normal saline (DNS) were administered daily to maintain fluid balance.

To combat infection and ensure proper wound healing, the patient received Inj. Taxim (cefotaxime) and Inj. Metrogyl (metronidazole) intravenously, both twice daily, along with Inj. Gentamicin (GM) and Inj. Rantac (ranitidine), both given twice daily. Additionally, the patient was provided with para IV infusion (paracetamol 1%w/v) and Inj. tramadol (50 mg/ml) intramuscularly, both administered twice daily for pain management. In cases of acute discomfort, Inj. Fortwin (pentazocine) and Inj. Phenergan (promethazine) were administered intramuscularly as needed.

Baby notes

On January 21, 2020, at 2:21 pm, a male infant was born, demonstrating signs of vitality and robust health. The baby was delivered at full term, indicating a gestational age of 40 weeks. Baby weighed 3.1 kg at birth, suggesting a healthy birth weight. Immediately upon delivery, the infant exhibited vital lung function and initiated crying, indicating successful respiration and adaptation to extrauterine life.

Apgar scores, which were an essential assessment of the newborn's overall well-being, were recorded at one minute and five minutes post-birth. At one minute, the baby received an Apgar score of 8 out of 10, signifying excellent physical and neurological responses. By the five-minute mark, the Apgar score had improved to 9 out of 10, indicating further improvement in the baby's condition, with near-perfect scores in various criteria such as heart rate, respiratory effort, muscle tone, reflex irritability, and skin color.

Discharge summary

It is essential for healthcare providers to ensure a smooth transition from IV to oral medications, providing clear instructions to the patient or their caregivers on how to continue the treatment at home. Transitioning from IV to oral medications helps reduce the risk of complications associated with prolonged IV therapy, such as infections, thrombosis, or other catheter-related issues. The patient's pain and discomfort were addressed through a regimen (depicted in Table 3) that included Combiflam tablets (ibuprofen + paracetamol), Omez capsules (omeprazole), Spade tablets (serratiopeptidase), T-Bact ointment (mupirocin 2.0%w/w) for external application, Cefixime tablets, Metrogyl tablets (metronidazole), Rantac tablets (ranitidine), and Paracetamol tablets, all carefully prescribed and administered according to the specified dosages and frequencies. This thorough medication regimen guaranteed the patient's comfort and well-being in the aftermath of surgery. 

**Table 3:** Drug Treatment P/O: Per oral, IM: Intramuscular; E/A: External application; OD: Once daily; BD: Twice daily; TDS: Three times daily; STAT indicates a one-time immediate dose * denotes the days on which the medication is scheduled

Brand Name	Generic name	Dose	ROA	Frequency	Duration with Dates							
					21/1	22/1	23/1	24/1	25/1	26/1	27/1	28/1
Tab. BCT	B-Complex	1 Tab.	P/O	OD	*	*	*	*	*	*	*	*
Tab. FST	Ferrous Sulphate	1 Tab.	P/O	OD	*	*	*	*	*	*	*	*
Tab. Calcium	Calcium	1 Tab.	P/O	OD	*	*	*	*	*	*	*	*
IV Fluid Ringer's Lactate	Ringer's Lactate	1 Pint	IV	OD	*				2*			
IV Fluid NS	Normal Saline	2 Pint	IV	OD	*							
IV Fluid DNS	Dextrose Normal Saline	1 Pint	IV	OD	*							
Inj. Taxim	Cefotaxime	1 g	IV	BD	*	*	*	*	*			
Inj. Metrogyl	Metronidazole	500 mg	IV	BD	*	*	*	*	*			
Inj. GM	Gentamicin	80 mg	IV	BD	*	*	*	*	*			
Inj. Rantac	Ranitidine	50 mg	IV	BD	*	*						
Inj. Para IV Infusion	Paracetamol	1%w/v	IV	BD	*	*						
Inj. Tramadol	Tramadol	50 mg/ml	IM	BD	*	*						
Inj. Fortwin	Pentazocine	30 mg/ml	IM	STAT	*							
Inj. Phenergan	Promethazine	25 mg/2ml	IM	STAT	*							
Tab. Combiflam	Ibuprofen + Paracetamol	400 mg + 325 mg	P/O	BD			*	*	*			
Cap. Omez	Omeprazole	20 mg	P/O	BD			*	*	*			
Tab. Spade	Serratiopeptidase	5 mg	P/O	TDS			*	*	*	*	*	*
T-Bactointment	Mupirocin	2.0%w/w	E/A	OD					*	*	*	*
Tab. Cefixime	Cefixime	200 mg	P/O	BD						*	*	*
Tab. Metrogyl	Metronidazole	500 mg	P/O	TDS						*	*	*
Tab. Rantac	Ranitidine	150 mg	P/O	BD						*	*	*
Tab. Paracetamol	Paracetamol	500 mg	P/O	BD						*	*	*

## Discussion

MAS is a significant cause of respiratory distress in newborns and can affect both full-term and post-mature infants. It is noteworthy that approximately 10-13% of healthy newborns may exhibit meconium staining in the amniotic fluid, and of these cases, approximately 4% may develop respiratory distress because of meconium aspiration. This underscores the importance of early recognition and appropriate management of this condition in neonatal care. In this case, we have discussed the pathophysiological complications associated with MAS, which primarily involve airway obstruction and inflammation. Meconium, when aspirated into the respiratory system, can lead to severe respiratory distress and potential long-term complications for the newborn. Prompt and effective intervention is crucial in mitigating the adverse outcomes associated with MAS.

One of the critical aspects of this case report was the use of prophylactic antibiotics in women undergoing LSCS. The timing of antibiotic administration for caesarean sections has been a subject of debate and research, with a focus on using the shortest and most appropriate regimen. The primary goal of administering antibiotics in the context of caesarean sections is to prevent post-operative infections, which can be a potential complication of these surgical procedures. It is also essential to consider the potential impact on the newborn, as the antibiotics administered to the mother may have implications for the neonate. In this case, a combination of antibiotics was employed to provide comprehensive coverage against potential pathogens. Cefotaxime, a third-generation cephalosporin, was used to target a broad spectrum of potential pathogens. Metronidazole was included to address infections caused by anaerobic organisms in the genital tract and abdominal cavity. Gentamicin, an aminoglycoside antibiotic, was added due to specific concerns for Gram-negative bacterial infections. The selection and administration of these antibiotics aim to reduce the risk of post-operative infections, ensuring the well-being of both the mother and the neonate.

Pain management is another critical aspect of the case, as it plays a significant role in the overall comfort and recovery of patients undergoing LSCS. The choice of pain management medications was individualized, taking into consideration the patient's needs, medical history, and recommendations from the healthcare team. Paracetamol was used for mild to moderate pain, both before and after surgery, due to its safety profile during pregnancy. Tramadol and pentazocine were employed as options for post-operative pain relief. This tailored approach to pain management reflects the importance of personalized care in maternal and neonatal health.

Furthermore, in this case, supplements and medications were administered to address specific health needs. B-complex and ferrous sulphate were provided to aid healing and manage anemia. Calcium supplementation was employed to maintain blood calcium levels, while Ringer's lactate and NS were used to provide essential fluids and electrolytes. DNS was administered to ensure energy and hydration, which are crucial aspects of the post-operative recovery process.

The transition of medications from IV or topical forms to oral administration in this discharge summary is a positive development that reflects the patient's progress toward recovery and the shift to more convenient and less invasive forms of treatment. Combiflam is a combination of ibuprofen and paracetamol. It was prescribed for pain relief following surgery. It can help reduce pain and inflammation. Omez is a brand name for omeprazole, a proton pump inhibitor. It is often prescribed to reduce stomach acid production and prevent or treat gastric issues, which may be necessary after surgery to prevent gastritis or heartburn. T-Bact is an antibiotic ointment containing mupirocin. It was used for wound care and to prevent or treat skin infections at the surgical site. Cefixime and metronidazole are antibiotics. They were prescribed for post-operative infection prevention or treatment. Effective management of MAS, careful consideration of antibiotic use in LSCS procedures, individualized pain management, and tailored supplementation collectively helped in achieving the goals of the therapy.

## Conclusions

This case highlights the importance of a multidisciplinary approach to maternal and neonatal care during and after LSCS. Properly timed and targeted antibiotic prophylaxis, individualized pain management, and the administration of supplements to address specific health needs contribute to a comprehensive and effective strategy for the well-being of the mother and the newborn. This case underscores the significance of personalized care in obstetrics and neonatal medicine and encourages further research and discussion on best practices in maternal and neonatal healthcare.
